# Assessment of the association between non-suicidal self-injury disorder and suicidal behaviour disorder in females with conduct disorder

**DOI:** 10.1186/s12888-021-03168-4

**Published:** 2021-03-26

**Authors:** Monika Szewczuk-Bogusławska, Małgorzata Kaczmarek-Fojtar, Agnieszka Adamska, Dorota Frydecka, Błażej Misiak

**Affiliations:** 1 Department of Psychiatry, Wroclaw Medical University, Katedra i Klinika Psychiatrii UM, Wybrzeże L. Pasteura 10, 50-367 Wrocław, Poland; 2 Department of Emergency Medicine, Wroclaw Medical University, Wrocław, Poland

**Keywords:** Depressive symptoms, Aggression, Anxiety, Suicide attempts, Conduct disorder

## Abstract

**Background:**

Non-suicidal self-injury (NSSI) and aggression have been demonstrated to serve as risk factors of suicidal behaviours (SB). Non-suicidal self-injury disorder (NSSID) and Suicidal Behaviour Disorder (SBD) are among new diagnostic categories for further studies in the DSM-5 classification.

**Methods:**

We recruited 196 girls (aged 15.5 ± 1.2 years) diagnosed with conduct disorder (CD). All of them were assessed with respect of non-suicidal self-injury acts, suicidal attempts, psychopathology, self-esteem and general functioning.

**Results:**

Age of NSSI onset was significantly lower compared to age of first suicidal attempt. SBD was present in 50.0% of patients with NSSID and the prevalence of NSSID in individuals with SBD was estimated at 52.2%. A diagnosis of NSSID, with at least 8 days of engagement in self-injuries during the preceding year, significantly predicted the risk of SBD. This effect appeared to be independent of depressive symptomatology.

**Limitations:**

Our results cannot be generalized over the whole population of individuals diagnosed with CD because of a lack of male patients, as well as individuals with the most severe and mildest forms of CD. Causal inferences cannot be established due to a cross-sectional study design.

**Conclusions:**

The NSSID with at least 8 days of engagement in self-injuries during the preceding year serves as a predictor of SBD independently of the effects of depressive symptoms. Longitudinal studies are required to confirm our findings.

**Supplementary Information:**

The online version contains supplementary material available at 10.1186/s12888-021-03168-4.

## Highlights

1. NSSID with modified frequency criterion predicts SBD in females with CD.
2. Current depressive symptoms serve as a predictor of SBD in girls with CD.
3. Total number of NSSI acts does not predict SBD in females with CD.
4. Anxiety, aggression and functioning are not relevant as predictors of SBD in CD.

## Introduction

According to the World Health Organization, suicide is the second leading cause of death in females aged 15–19 years and is ranked as the third cause of death among men of the same age [1]. Epidemiological data from the United States show that suicide is also the second cause of death in two groups of youth: children (aged 10–14 years) as well as adolescents and young adults (aged 15–24 years) [2]. At present, risk factors of suicide are being intensively researched in the general population of adolescents as well as in various clinical populations. Non-suicidal self-injuries (NSSIs) occur in 17–18% of adolescents in community samples [3], and though they are not related to suicidal ideation, they lead to body tissues’ damage. The common feature of NSSIs and suicidal behaviour (SB) is the destruction of own body, which potentially places NSSI close to SB. The link between NSSIs and suicidal behaviour (SB) has previously been postulated in both theoretical models and empirical studies, suggesting that NSSI acts as one of the crucial risk factors of SB [4].

The gateway theory explains the relationship between NSSI and SB as a continuum with NSSI and fatal suicide at the ends of the same spectrum of behaviours. This theory is based on the following facts: 1) according to epidemiological data, NSSIs triple the risk of SB; 2) the onset of NSSIs precedes the onset of SB and 3) NSSIs remain a highly predictive risk factor after adjustment for other potential risk factors. Based on these arguments NSSIs are suggested a gateway towards SB [5–7]. Another theoretical model assumes the presence of other factors (third variables) that link NSSIs and SB. According to the third variable theory, independent factors or other variables affect the association between NSSIs and SB. Low self-esteem, depressive symptoms, personality disorders, suicidal ideation and low familial support are considered as crucial third variables. Due to clinical and theoretical limitation of the described theories, integrated models were proposed [8–10]. The Joiner’s integrated model adds, neuroscience-derived pain modulation as a variable to the existing continuum [11]. Taking into account the strong relationship between the number of NSSIs and the risk of SB, the authors suggest that NSSIs lead to desensitisation of pain, reducing fear and eventually enable suicidal acts. The integration model has been developed by other authors; severity of NSSIs, violent experience in the past, alcohol or drug exposure are among factors affecting the association between NSSIs and SB. Furthermore, NSSIs are postulated as an emotional regulation strategy, if this strategy fails, an individual engages in more severe acts that are closer to suicide [12].

Searching for risk factors and predictors of suicide, both NSSIs and SB have extensively been investigated in adolescence. Aggression was among the variables suggested to play a role in the association between NSSIs and SB. The results of the latest follow-up study in adolescents with suicide risk revealed five profiles of elevated risk. Apart from severe suicidal ideations and behaviour as well as a history of suicidal thoughts and behaviours, aggression appeared to be a significant predictor of suicide in two profiles [13]. In other studies, aggression (verbal and physical) was identified as one of risk factors of suicidal ideation, attempts and non-suicidal self-injuring behaviours [14–17]. Furthermore, antisocial personality traits, which are linked to violent behaviours and aggression, were shown to predict NSSI in young adults [18]. Antisocial personality is preceded by conduct disorder (CD) in adolescence. CD is characterized by a number of aggressive behaviours that violate the basic rights of others and/or societal norms or rules. Physical aggression to people or animals, destruction of property, deceitfulness or theft, serious violations of rules are also the crucial symptoms of CD. The association between self-injury, suicidal behaviours and CD has also been suggested based on previous results [19, 20].

Although in the last decade, growing body of evidence supports the relationships between NSSIs and SB, a meta-analysis of 172 studies revealed weaker than expected effect of prior self-injurious thoughts and behaviors as a risk factor of suicide [21]. It is worth noting that in the majority of previous studies, NSSIs have not been classified according to specific and unified criteria. After the development of DSM-5 criteria by members of the International Society for the Study of Self-Injury and their introduction by the American Psychiatric Association [22], NSSIs as well as suicidal attempts are classified as separate psychiatric diagnoses and are referred to as non-suicidal self-injury disorder (NSSID) and suicidal behaviour disorder (SBD). Both diagnostic categories are listed as conditions that require further research to investigate their validity.

On the basis of current DSM-5 criteria, Groschwitz et al. found high co-occurrence of NSSID and SBD in hospitalized adolescents. More than half of individuals (51.2%) with NSSID met the SBD criteria in a sample of psychiatric inpatients, and 65.6% of individuals diagnosed with SBD met the NSSID criteria [23]. However, a growing body of evidence from previous studies suggest the need of reconsideration of NSSID criteria proposed in the DSM-5. Primarily, the research focusing on the frequency criterion of NSSID shows the need of revision. Results indicate that an increase to 8, 10 or even 25 days of self-injuring episodes during the preceding year should be considered. The criterion of at least 8 days was found as a threshold of NSSIs in females with CD from our previous study [24]. Ten or more days of engagement in NSSIs was indicated as the threshold frequency criterion based on the results in a community sample [25]. The threshold of 25 or more days was identified in adolescent inpatients who participated in treatment programs for individuals with NSSI or SB [26].

From a clinical perspective, assessment of suicide risk serves as the key point in psychiatric decision-making process. Thus, the relationship between NSSID, concomitant psychopathology and SBD should be clearly defined to create diagnostic and therapeutic guidelines for specific mental disorders in order to predict life-threatening behaviours. Given that NSSIs, depression and aggression are risk factors for SB, our aim was to find a homogenous sample of participants in terms of psychiatric diagnosis with high prevalence of NSSIs on the one hand, as well as common and mixed psychopathology on the other hand. In our previous study [24], we showed that more than 50% of girls with CD engage in NSSIs during their lifetime. Taking into account that individuals with CD present two well-established risk factors (aggression and NSSIs), we decided to conduct our study in this specific group to identify more precisely the specificity of those factors. To the best of our knowledge, the association between NSSIs and SB in patients diagnosed with CD on the basis of DSM-5 criteria or new frequency criteria postulated in previous studies has not been reported so far. Therefore, we tested the hypothesis whether a diagnosis of NSSID, according to the higher frequency threshold determined by our group [24] predicts a diagnosis of SBD. In addition, we hypothesised that SBD is related to current concomitant depressive and anxiety symptoms, the level of aggression and general functioning in patients with CD.

## Methods

One hundred ninety-six girls (15.5 ± 1.2 years) who were residents of the Youth Sociotherapy Center (YSTC) No. 2 in Wrocław (Poland) were enrolled. YSTCs are Polish facilities created for children and adolescents with educational, developmental problems or CD who are at risk of social maladjustment. Residents of YSTC receive schooling, accommodation and sociotherapy. Individuals are recruited to the facility on the basis of pedagogical and psychological opinion. Standard psychological procedures including WISC-R and/or IDS- 2 test were used to exclude individuals with intellectual disability.

During first in-person meeting, an investigator (psychologist or child and adolescent psychiatrist) explained and discussed all study procedures with the examined person. Then, after reading the paper version of the consent (compliant with the requirements of the Bioethics Committee) the patient signed and dated the form in the presence of the researcher. After consenting, all participants were interviewed by trained psychologists or child and adolescent psychiatrists to obtain the information regarding demography, family, medical and psychiatric history. To establish the diagnosis of CD, a psychologist or child and adolescent psychiatrist examined all participants. In addition, the CD and the Suicidal Tendencies Sections of the Polish version of the Mini International Neuropsychiatry Interview for Children and Adolescent (MINI-KID) were administered to confirm the diagnosis and assess the frequency, the number, the reasons and the age of the first and the last suicidal attempts [27]. In the next step, information regarding frequency, recency, methods and functions of NSSIs was obtained. Semi-structured interview was administered by clinicians to collect the data. In the third part, participants were asked to fulfil a set of questionnaires to collect data regarding the level of concomitant psychopathology (depressive symptoms, anxiety, aggression, self-esteem and functioning). The following measures were used: the Global Assessment of Functioning (GAF), the Buss–Perry Aggression Questionnaire (BPAQ), the Children’s Depression Inventory (CDI), the Spielberg State-Trait Anxiety Inventory (STAI) and the Rosenberg Self-Esteem Scale (SES) [28–33].

The study protocol was approved by the Ethics Committee and all participants and their parents or legal caregivers gave an informed consent for participation.

### Measures

#### Mini-kid

The MINI-KID [32] is a structured diagnostic interview designed to assess the symptoms of mental disorders according to the *DSM-IV* and *ICD-10* criteria in children and adolescents aged 6 to 17 years. The MINI-KID can be administered by interviewing exclusively adolescent respondents or by interviewing both the child/adolescent and the parent(s) or caregivers. Good psychometric properties of the MINI-KID were shown in the study of the Polish version with comparable validity parameters to those obtained by other studies [27].

#### NSSIs - interview

As part of a psychiatric examination, we created a semi-structured interview to collect the information about the age of onset, number, methods and purposes of NSSI, using NSSI-semi -structered interview (Amendment):

The diagnosis of NSSID was established if the following answers were obtained:

1. NSSID criterion A:

- “Yes” to question 1 and “No” to question number 2;
- At least one of following: cutting, burning, picking, hitting and/or excessive rubbing indicated as the answer to question number 3.
- At least 8 days during last year answered to question number 9.

2. NSSID criterion B:

- One of the following: relieving an interpersonal difficulty, reducing negative emotions and inducing positive feelings answered to question number 5 (open or in subsection statements).

3. NSSID criterion C:

- One of the following answers to open questions number 4 or 5: Interpersonal problems (in relationships with family members or friends) or negative thoughts or emotions immediately before NSSI, preoccupation with NSSI that is difficult to manage, persistent and /or frequent thoughts about NSSI.

4. We assessed Criterion E (the functioning impairment) based on GAF.

#### Global assessment of functioning (GAF)

The GAF is a scale designed to assess severity of mental disorders. The scale is divided into 10 sections (each interval describes specificity of functioning in different areas). Scoring might be conducted on the basis of the following information sources: medical history and medical records, scales, questionnaires and other reports provided by clinicians, caregivers or parents [33]. In our study, assessment with the GAF was based on information from many sources (participants, teachers and caregivers). The GAF scoring was performed by the same rater for all individuals.

#### Children depression scale (CDI)

This measure consists of 27 items and allows to assess depressive symptoms in children and adolescents aged 7–17 years. The questionnaire includes subscales measuring depressive mood, interpersonal difficulties, ineffectiveness, anhedonia and negative self-esteem [29]. The Cronbach’s alpha for CDI was 0.94 in our sample.

#### STAI

The STAI is a tool which contains two subscales for assessing state (X1) and trait anxiety (X2). State anxiety (X1) is referred to as emotional status which is variable and dependant on circumstances, and trait anxiety (X2) that is stable and depends on individual’s personality in terms of experiencing emotions. The measure consists of 40 items and is rated on a scale scored between 1 and 4 [31]. The Cronbach’s alpha was 0.94 for state anxiety and 0.99 for trait anxiety.

#### SES

The SES is a self-rated instrument for global self-esteem assessment. It measures positive and negative feelings about the self. The questionnaire consists of 10 statements that are based on a 4-point Likert scale. The person completing the scale may strongly agree, agree, disagree or strongly disagree with 10 statements regarding their self-worth [30]. The Cronbach’s alpha for the SES total score was 0.89.

#### BPAQ

The BPAQ is a self-report rating questionnaire which consists of 29 items and is scored on a 5-point Likert- type scale (1- “extremely uncharacteristic of me” to 5 - “extremely characteristic of me”). A range of aggressive symptoms are measured by specific subscales (physical aggression, verbal aggression, anger and hostility) [28]. The Cronbach’s alpha for the BPAQ total score in our sample was 0.80, for physical aggression 0.77, for verbal aggression 0.73, for anger 0.62 and for hostility 0.77.

#### Data analysis

Normality of data distribution was assessed using the Kolmogorov-Smirnov test. Before further analyses, participants were divided into two subgroups – individuals who met the SBD criteria and those who did not (SBD(+) and SBD(−) adolescents, respectively). In case of non-normal distribution (age, educational delay, STAI – trait anxiety, BPAQ – hostility, age of first suicidal attempts, age of NSSIs onset, lifetime and one-year number of NSSI acts and suicidal attempts) the comparison of continuous variables between SBD(+) and SBD(−) adolescents was performed using the Mann-Whitney U test. Otherwise, independent sample t-tests were performed (scores of GAF, CDI, STAI – state anxiety, BPAQ – physical aggression, BPAQ – verbal aggression, BPAQ – anger and SES). Distribution of categorical variables (place of prior residence, medication status, severity of CD and the level of intelligence) was compared using the χ^2^ test. Due to multiple bivariate comparisons, the Bonferroni correction was applied to the level of significance. Therefore, results of bivariate comparisons were considered statistically significant if the *p*-value was less than 0.0023. All variables significantly associated with a diagnosis of SBD after adjustment for multiple testing were subsequently analysed using the binary logistic regression to test their independent effects. A diagnosis of SBD was included as the dependent variable. To avoid potential multicollinearity, separate models, testing for the effects of lifetime number of NSSIs acts, recent-year number of NSSIs acts and a diagnosis of NSSID (according to the new frequency criterion assessed in our previous study), were analysed. The Nagelkerke R^2^ was used to assess the model fit. The alpha criterion level was set at 0.05 in binary logistic regression analysis.

## Results

General characteristics of adolescents recruited in this study with respect to a diagnosis of SBD are shown in Table 1. Both groups did not differ significantly in terms of age, place of prior residence, educational delay, intelligence level, CD severity and medication status after adjustment for multiple testing. Participants from the SBD(+) group were younger and were more frequently medicated (trend level significance) in comparison with SBD(−) individuals. The age of the NSSIs onset was significantly lower compared to the age of the first suicidal attempt (*p* < 0.001). The prevalence of SBD in adolescents with NSSID was 50.0%. In turn, the prevalence of NSSID in the group of SBD(+) individuals was estimated at 52.2%.

**Table 1:** General sample characteristics

	SBD(+), *n* = 46	SBD(−), *n* = 150	p
Age	15.1 ± 1.1	15.6 ± 1.1	0.008
Place of prior residence			
- Educational care facility	9	22	0.252
- Home	28	114	
Educational delay, years	1.1 ± 0.8	1.3 ± 0.8	0.252
Intelligence			
- Below-average	1	3	0.640
- Normal	35	120	
- Above-average	2	14	
Conduct disorders severity:			
- Mild	24	83	0.686
- Moderate	14	36	
- Severe	8	30	
Medication status:			
- Medicated	11	17	0.012
- Unmedicated	27	122	

The comparison of psychopathology, general functioning and NSSI characteristics between SBD(+) and SBD(−) adolescents is presented in Table 2. SBD(+) adolescents had significantly higher levels of depressive symptoms, trait and state anxiety, recent-year and lifetime number of NSSI acts. A diagnosis of NSSID, according to the frequency criterion proposed in our previous study, was significantly more frequent in SBD(+) adolescents compared to those from the SBD(−) group. The GAF and SES scores were also significantly lower in SBD(+) individuals.

**Table 2:** Bivariate analyses of factors associated with SBD diagnosis

	SBD(+), n = 46	SBD(−), n = 150	p
CDI (global score)	25.7 ± 11.0	14.7 ± 8.8	**< 0.001**
STAI-X1	46.9 ± 10.7	39.8 ± 11.0	**< 0.001**
STAI-X2	52.1 ± 9.3	45.0 ± 10.5	**< 0.001**
BPAQ-PA Physical Aggression	19.7 ± 8.2	17.5 ± 8.1	0.191
BPAQ-VA Verbal Aggression	12.1 ± 4.7	11.6 ± 3.8	0.398
BPAQ-A Anger	16.9 ± 6.1	14.1 ± 5.6	0.022
BPAQ-H Hostility	20.1 ± 7.7	18.3 ± 6.2	0.163
SES	23.2 ± 5.6	27.7 ± 5.8	**< 0.001**
GAF	54.6 ± 14.0	64.8 ± 11.7	**< 0.001**
NSSID (yes/no)	24/22	24/122	**< 0.001**
Recent-year number of NSSI acts	21.8 ± 28.4	8.5 ± 24.8	**< 0.001**
Lifetime number of NSSI acts	95.4 ± 197.4	29.3 ± 106.0	**< 0.001**
Age of NSSI onset	12.5 ± 1.8	13.1 ± 1.4	0.023
Age of first suicidal attempt	13.7 ± 1.4	13.5 ± 1.3	0.619
Means of NSSI, number	1.8 ± 1.2	1.2 ± 0.6	0.011

Significant differences after Bonferroni correction (*p* < 0.0023) were marked with bold characters

Three distinct binary logistic regression models were tested (Table 3). Variables associated with a diagnosis of NSSID were tested in separate models to avoid multi-collinearity. Model 1 had the highest Nagelkerke R^2^ statistics (0.309) and the percentage of correctly classified individuals (81.0%). There were significant independent effects of depressive symptoms on the risk of SBD in all three models (model 1: OR = 1.079, 95%CI = 1.009–1.155, *p* = 0.027; model 2: OR = 1.093, 95%CI = 1.022–1.169, *p* = 0.009; model 3: OR = 1.093, 95%CI = 1.023–1.168, *p* = 0.009). A diagnosis of NSSID, according to the operationalization proposed by our group, significantly predicted the risk of SBD (OR = 2.914, 95%CI = 1.073–7.917, *p* = 0.036). However, the recent-year (OR = 1.002, 95%CI = 0.988–1.016, *p* = 0.761) and lifetime number of NSSI (OR = 0.570, 95%CI = 0.998–1.004, *p* = 0.570) acts was not significantly associated with the risk of SBD. Similarly, trait and state anxiety, self-esteem and general functioning did not appear to be significant predictors of SBD.

**Table 3:** Factors associated with the diagnosis of SBD in a binary logistic regression analysis

	Nagelkerke R^2^	Percentage of correctly classified subjects	Variable	B	S.E.	Wald	OR	95%CI	p
Model 1	0.309	81.0	STAI-X1	− 0.005	0.027	0.032	0.995	0.944–1.049	0.859
			STAI-X2	−0.003	0.030	0.008	0.997	0.941–1.057	0.927
			CDI	0.076	0.035	4.871	1.079	1.009–1.155	**0.027**
			SES	−0.001	0.054	0.001	0.999	0.899–1.109	0.982
			GAF	0.019	0.019	2.344	0.971	0.935–1.008	0.126
			NSSID	1.070	0.510	4.401	2.914	1.073–7.917	**0.036**
Model 2	0.273	79.6	STAI-X1	−0.006	0.026	0.058	0.994	0.944–1.046	0.809
			STAI-X2	−0.006	0.029	0.037	0.994	0.939–1.054	0.848
			CDI	0.089	0.034	6.775	1.093	1.022–1.169	**0.009**
			SES	−0.011	0.054	0.045	0.989	0.890–1.099	0.832
			GAF	−0.037	0.019	3.807	0.964	0.929–1.000	0.051
			Recent-year’s number of NSSIs	0.002	0.007	0.093	1.002	0.988–1.016	0.761
Model 3	0.275	80.3	STAI-X1	−0.008	0.026	0.099	0.992	0.942–1.044	0.752
			STAI-X2	−0.004	0.029	0.015	0.996	0.941–1.055	0.902
			CDI	0.089	0.034	6.859	1.093	1.023–1.168	**0.009**
			SES	−0.011	0.054	0.040	0.989	0.890–1.099	0.842
			GAF	−0.035	0.019	3.358	0.965	0.930–1.002	0.067
			Lifetime number of NSSIs	0.001	0.001	0.322	0.570	0.998–1.004	0.570

Significant effects were marked with bold characters (*p* < 0.05)

## Discussion

Our main finding shows that NSSID with a modified criterion A (8 or more days of engagement in NSSI) serves as an independent risk factor of SBD in female individuals with CD. However, the number of NSSIs was not found to be associated with the SBD risk. To the best of our knowledge, the data regarding the relationship between NSSID and SBD diagnoses in adolescents was published in only one paper [22]. The authors recruited 111 inpatients diagnosed with affective (53.2%), anxiety or stress-related disorders (31.5%), behavioural and emotional disorders (20.7%) and eating disorders (18%). Based on the results, they concluded that female gender and a diagnosis of affective disorders serve as risk factors for both NSSID and SBD. Moreover, NSSIs were shown to be a strong risk factor of SBD in the inpatients’ sample. Although our sample characteristics were different compared to those in the study by Groschwitz et al. [23], our results replicate some findings. Indeed, we also found significantly lower age of onset of NSSID in comparison with SBD. Additionally, NSSID was found to be an independent risk factor of SBD in our study. However, in our analyses, the diagnosis of NSSID was established based on the new NSSI frequency threshold validated in our previous study [24]. It is worth emphasizing that neither the lifetime number of NSSIs nor the number of NSSIs during the preceding year serve as predictors of SBD. Ghinea et al. suggest that NSSID as a stand-alone diagnosis is rare and unstable but showed that NSSIs are a precursor of the suicide attempt [34]. Our results support the utility of NSSID diagnosis (with modified frequency criterion) but not NSSIs, regardless of the number of acts, as a risk factor of SBD in this specific clinical setting and are in line with the results obtained by Kiekens et al. [35] who found that subthreshold NSSID is less related to suicidality than NSSID with all the diagnostic criteria met.

In our study, every second girl diagnosed with current NSSID diagnosis attempted suicide during the preceding 2 years and met the SBD criteria. Although there is a scarcity of studies addressing the relationship between NSSID and SBD, analyses of the association between NSSID with the new frequency criteria and suicidality have been performed previously. Results of the study in a community sample, which included 10 or more NSSIs as the threshold frequency criterion, showed that individuals who had met the new criterion were significantly different than the subthreshold group (less than 10 acts of NSSI) and controls (those with a negative history of engagement in NSSIs) in terms of lifetime suicide attempts with the following prevalence estimates: 20.4, 2.9 and 0.7%, respectively. Similar differences between those three groups were observed regarding suicidal ideations [25]. Data from another study by Muehlenkamp et al. [26] that was based on adolescent inpatients who were divided into three groups depending on the number of NSSIs during the preceding year (low: 1–4 NSSIs, moderate: 5–24 NSSIs and high: 25 or more NSSIs) showed that the “high-frequency group” was significantly different from both „moderate-” and „low-” frequency groups in terms of NSSIs and suicidal ideation and planning suicide during the preceding year, but not with respect to lifetime suicide attempt risk. Prevalence rates of lifetime suicide attempts were estimated at: 39.0% (in „the low-frequency group”), 42.3% (“the moderate-frequency group”) and 48.7% (in “the high-frequency group”). The majority of patients from this study were diagnosed with mood disorders and all individuals participated in special treatment programs for adolescents with NSSIs or suicidal behaviours.

The studies described above differ significantly from our study in terms of methodological aspects. These studies analysed lifetime suicide attempts in contrast to the preceding 2 years (according to SBD criteria) in our study. Our results show very high risk of suicide attempt (50% girls met the SBD criteria) in females with CD and concomitant NSSID diagnosis (with 8 days of engagement in NSSIs) in comparison with both community samples and adolescent inpatients. The two-year prevalence of suicide attempts in our sample was higher than the lifetime prevalence in hospitalized patients with the most severe NSSIs [26].

We also found that 52.2% individuals with SBD meet the NSSID criteria. The coincidence of these diagnoses might be considered relatively rare but it is disputable due to the time criterion and cross-sectional methodology. The diagnosis of SBD is based on a two-year frequency, while the frequency during the preceding year is included as the diagnostic criterion of NSSID. Therefore, patients could meet NSSID criteria at the time of attempting suicide but did not meet them at the time of examination during the study. Thus, further follow-up studies are needed to assess comorbidity of SBD and NSSID.

Moreover, we showed that current depressive symptoms also serve as an independent predictor of SBD in girls with CD. The comorbidity of CD and depressive disorders have been documented by several studies that included individuals with CD [36–38]. The co-occurrence of depressive symptoms clearly places CD among mental disorders with increased risk of suicide. Our findings are in line with results from previous studies, suggesting that depression or mood symptoms appear to be mediators in the relationship between CD and suicidal behaviours [19, 39, 40]. Additionally, previous Finish birth cohort studies revealed an increased risk of suicidal behaviours in patients, demonstrating both CD and mood disorders symptoms [19]. Similarly, Vander Stoep et al. [40] assessed a risk of suicidal behaviours in adolescents during a 2-year follow-up period and found the highest risk of suicidal ideation, suicide attempts and recurrent suicidal behaviour in patients who presented both depression and CD symptoms. Our results indicate the need of screening girls with CD for depressive symptoms. Identification of individuals who might be at risk of depression should be recommended in adolescents with CD.

Interestingly, we did not find that aggression is a risk factor of SBD in girls with CD. Even though, Wei et al. [20] found that CD is independently related to an increased likelihood of attempting suicide, little is known about the role of aggression in patients with SBD and CD. The authors showed the relationship between CD and suicide attempts that remained significant after controlling for a diagnosis of depression and substance use disorders [20]. These findings were replicated by a longitudinal study of a population-based cohort of adolescents [41]. However, the authors did not assess the role of aggression level in SBD. Our data does not indicate the effect of aggression, regardless of its type, on SBD risk. Individuals diagnosed with CD present a broad spectrum of negative emotions, including anger and hostility, which may lead to aggressive behaviours. However, this particular pattern of aggressive behaviours differentiates this group from other disorders and our findings show that the level of aggression in CD is not associated with the increased risk of SBD. In other words, regardless of the severity of aggressive behaviours, individuals with CD are at very high risk of suicide attempts and even mild conduct problems might lead to a suicide attempt. On the other hand, it is worth mentioning that our sample was limited to females and thus generalization of findings to the whole population of adolescents cannot be made.

To the best of our knowledge, this is the first study which demonstrates the relationship between NSSID and SBD in CD on the basis of DSM-5 criteria. However, the present study has certain limitations that need to be discussed. First, it should be noted that we did not include males into our sample, as well as individuals with the most severe forms of CD (who are hospitalized due to concomitant mental disorders or are residents of detention juvenile centers) or mildest forms of CD (who are treated in outpatient clinics) and thus our results cannot be generalized over the whole population of individuals diagnosed with CD. These limitations may also have an impact on the lack of association between aggression and SBD. Assessment of the effect of aggression should be for both genders taking into account the severity of CD as well as the comparison with control groups from community samples or individuals diagnosed with other mental disorders. Other limitations apply to the tools we used. We did not use any instrument to establish concomitant psychiatric diagnoses. Assessment of comorbidity seems to be crucial to provide more information regarding the effect of psychopathology. It should also be added that all characteristics of NSSID were based on an assessment using a semi-structured interview due to non-availability of the Polish validation of diagnostic instruments. We also used the GAF with questionable psychometric parameters that was excluded from the DSM-5. However, the data was gathered from many sources of information to assess patients’ functioning and was performed by the same rater. It is also important to note that a cross-sectional study design does not allow to provide insights into direction of causality. A cross-sectional design also does not allow to assess temporal patterns of the development of SBD and the role of mediating and moderating variables. The percentage of variance explained by our binary logistic regression model that included NSSID as a predictor of SBD was relatively low (30.9%). This observation indicates that there are also other predictors of SBD in adolescents with CD that were not recorded in the present study. Therefore, future studies should consider other variables or factors to address the association between NSSID and SBD in this specific disorder. Indeed, comorbidity, functions of NSSIs, familial or social support, history of early-life stress and specific personality traits may also act as predictors of SBD in CD.

In summary, our results indicate that the diagnosis of NSSID with a higher frequency threshold of NSSI acts is a risk factor of SBD, regardless of the independent effect of depressive symptoms in female adolescents with CD. However, longitudinal studies in larger samples of females and males with CD are required to confirm and indicate direction of causality. Our findings may allow to differentiate the group with high risk of suicide among females diagnosed with CD. Due to the fact that the analysis of the onset of disorders showed that NSSID precedes SBD, the implementation of NSSIs prevention programmes may contribute to a reduction of the frequency of suicide attempts in this group of adolescents.

## Supplementary Information

**Additional file 1.** NSSI – semi-structered interview.

## Data Availability

Data will be available upon reasonable request sent to the corresponding author.
