# Supplementary material for: Assessment of the association between non-suicidal self-injury disorder and suicidal behaviour disorder in females with conduct disorder
Source: BMC Psychiatry. 2021 Mar 26;21:172. doi: 10.1186/s12888-021-03168-4 (PMC7995747; doi:10.1186/s12888-021-03168-4)
Supplement: Supplementary file 1 — Additional file 1. NSSI – semi-structered interview. [file 12888_2021_3168_MOESM1_ESM.docx]

| Question | YES | NO | N/A | Comments: |
| --- | --- | --- | --- | --- |
| 1.Have you ever deliberately injured yourself ? |  |  |  |  |
| 2. Were you having any suicidal intention when you were doing this? |  |  |  |  |
| 3.What methods have you used to harm yourself? |  | | |  |
| 4.For what reasons have you injured yourself? |  |  |  |  |
| 5. For what purpose have you injured yourself? |  |  |  |  |
| to alleviate anxiety/sadness/tension |  |  |  |  |
| to manage bad feelings |  |  |  |  |
| to punish or control somebody |  |  |  |  |
| to get someone’s attention |  |  |  |  |
| to punish yourself |  |  |  |  |
| to feel better |  |  |  |  |
| 6. How old were you when you engaged in this behaviour for the first time? |  | | |  |
| 7. How many times have you done this since you started? |  |  |  |  |
| 8. Have you harmed yourself during the last year? |  |  |  |  |
| 9. How many days did you injure yourself last year? /last month?/last week? |  |  |  |  |
| 10. When did you injure yourself last time? |  |  |  |  |

NSSI – semi-structered interview
